# The Ensemble of Conformations of Antifreeze Glycoproteins (AFGP8): A Study Using Nuclear Magnetic Resonance Spectroscopy

**DOI:** 10.3390/biom9060235

**Published:** 2019-06-17

**Authors:** Cheenou Her, Yin Yeh, Viswanathan V. Krishnan

**Affiliations:** 1 Department of Chemistry, California State University, Fresno, CA 93740, USA; cheenouher09@mail.fresnostate.edu; 2 Department of Applied Science, University of California, Davis, CA 95616, USA; 3 Department Medical Pathology and Laboratory Medicine, Davis School of Medicine, University of California, Davis, CA 95616, USA

**Keywords:** AFGP, NMR, ensemble of structures, antifreeze proteins

## Abstract

The primary sequence of antifreeze glycoproteins (AFGPs) is highly degenerate, consisting of multiple repeats of the same tripeptide, Ala–Ala–Thr*, in which Thr* is a glycosylated threonine with the disaccharide *beta-d-galactosyl-(1,3)-alpha-N-acetyl-d-galactosamine.* AFGPs seem to function as intrinsically disordered proteins, presenting challenges in determining their native structure. In this work, a different approach was used to elucidate the three-dimensional structure of AFGP8 from the Arctic cod *Boreogadus* *saida* and the Antarctic notothenioid *Trematomus* *borchgrevinki*. Dimethyl sulfoxide (DMSO), a non-native solvent, was used to make AFGP8 less dynamic in solution. Interestingly, DMSO induced a non-native structure, which could be determined via nuclear magnetic resonance (NMR) spectroscopy. The overall three-dimensional structures of the two AFGP8s from two different natural sources were different from a random coil ensemble, but their “compactness” was very similar, as deduced from NMR measurements. In addition to their similar compactness, the conserved motifs, Ala–Thr*–Pro–Ala and Ala–Thr*–Ala–Ala, present in both AFGP8s, seemed to have very similar three-dimensional structures, leading to a refined definition of local structural motifs. These local structural motifs allowed AFGPs to be considered functioning as effectors, making a transition from disordered to ordered upon binding to the ice surface. In addition, AFGPs could act as dynamic linkers, whereby a short segment folds into a structural motif, while the rest of the AFGPs could still be disordered, thus simultaneously interacting with bulk water molecules and the ice surface, preventing ice crystal growth.

## 1. Introduction

Following initial reports on the discovery of antifreeze glycoproteins (AFGPs) by Scholander and coworkers [1,2], DeVries and Feeney [3,4] began to investigate the mechanisms of antifreeze action. AFGPs are a class of biological antifreeze that help fish in Arctic and Antarctic regions survive in supercooled water [3,5]. The biological basis of the function of AFGPs, explained with concepts involving freezing point depression and ice recrystallization inhibition, are well known [3,6,7]. AFGPs’ function is prominently displayed by *thermal hysteresis* behavior, where the freezing and melting points of the AFGP solution differ. Studies have also shown that AFGPs slightly increase the melting point of ice crystals, resulting in superheated ice crystals present at a temperature above the normal melting point of water [8,9]. With regard to the nature of how AFGPs interact with ice crystals, these proteins are known to alter the nature of the ice crystal morphology. Therefore, they are often considered to be a subset of ice-binding proteins (IBPs) [10]. When ice crystals form in the presence of AFGPs, the ice crystal morphology changes from the normal hexagonal-shaped planes into a needle, prism-like structure [11,12,13]. To date, AFGPs are the most effective ice recrystallization inhibitor, several times more potent than polyvinyl alcohol (PVA) [14,15].

Most research in the last decade has been focused on antifreeze proteins (AFPs), non-glycosylated antifreeze proteins, and several excellent reviews summarizing the progress in this area have been published [10,16,17,18,19,20,21,22,23,24]. In comparison, AFGPs have been less studied, in part because they do not form a well-defined three-dimensional structure, rendering it difficult to crystallize the protein system. In addition to their structural complexity, AFGPs are not amenable to overexpression by conventional molecular biology techniques in *Escherichia coli*, a general requirement for structural studies of complex proteins in a solution state using nuclear magnetic resonance (NMR) spectroscopy. One comprehensive review article that included an extensive discussion on AFGPs was by Yeh and Feeney [7]. In addition, several review articles have focused on one of the specific aspects of AFGPs. A review article by Harding and coworkers summarized the structural and physical properties of AFGPs, mainly based on studies in a solution state, with a particular emphasis on genetic evolution and biological applications [22]. A series edited by Graether [25] contained notable articles related to AFGPs, including one from some of the authors of this work [26]. More recently, Urbanczyk et al. reviewed the structure and activity relationship of AFGPs as well as an overview of the status of their chemical synthesis [27].

In a traditional sense, understanding the function of a protein is directly linked to the folded 3D structure of the protein, as in the case of AFPs. Since their discovery, there have been only two experimentally determined three-dimensional structures of AFGPs. The first natural AFGP structure was provided through high-resolution solution NMR experiments by Lane et al. [28,29]. These studies were done on both AFGP8-Pro (14 amino-acid residue Thr*-Pro-Ala glycoprotein AFGP8 from the Antarctic cod *Trematomus borchgrevinki*) and a mixture of AFGP fractions 1–5 (AFGP1–5). The modeled structures of AFGP8 in water determined by these investigators suggest that the protein lacks long-range order (an absence of a long-range nuclear Overhauser effect (NOE) or NOEs representative of the formation of a protein tertiary structure). However, there is evidence of local order involving the two –Ala–Thr*–Pro–Ala– segments. The second detailing structural data was on a synthetic AFGP (syAFGP_3_:–Ala–Thr*–Ala–Ala–Thr*–Ala–Ala–Thr*–Ala–) provided by Tachibana et al. [30]. Surprisingly, the synthetic AFGP appeared to form a well-defined structure, despite the absence of long- or medium-range constraints, supporting the analysis that the peptide backbone folds into a left-handed helix in which three-disaccharide moieties are on the same side of the molecule, thus constructing a hydrophilic face. In addition, it was discovered that the Ala-CH_3_ groups and acetyl methyl groups in the GalNAc moiety were clustered onto one side of the molecule, forming a hydrophobic face.

In the absence of high-resolution three-dimensional structural models, two important protein structure-based factors have emerged that might be relevant to understanding the function of AFGPs: The significance of Pro residues (composition and relative juxtaposition) and a possible definition of a local structural motif. To address these points, we developed a three-dimensional structural model of the smallest fraction: AFGP fraction 8 (AFGP8) from two different natural sources, the Arctic cod *Boreogadus saida* (AFGP8-BS) and the Antarctic notothenioid *Trematomus borchgrevinki* (AFGP8-TB), were studied in deuterated dimethyl sulfoxide (DMSO) using NMR spectroscopy. With the viscosity of DMSO at 25 °C (1.99 cP) [31], mimicking the viscosity of aqueous AFGP solution at 0 °C (~2.0 cP) [32], it was hypothesized here that DMSO could be a potential model solvent to investigate the ensemble of structures of AFGPs. In support of this hypothesis, we argue that the approach presented here is a method to generate model structures of AFGPs that might provide ideal native conditions. It has been demonstrated previously by Heisel and Krishnan [33] that solvent perturbation can be used to study the ordered-to-disordered transition of modeled FG-nucleoporin peptide, which is part of an intrinsically disordered peptide/protein network within the nucleopore complex [33]. In the case of AFGPs, which might function as effectors and entropic chains, AFGPs might make a disordered-to-ordered transition upon binding to the ice surface. The use of DMSO was somewhat like taking a “snapshot”, a conformation that was part of the dynamic ensemble of conformations of AFGPs in aqueous conditions. The “snapshot” conformation could then be studied in detail to see possible local interactions that are not transparent in AFGPs’ dynamic native conditions. Given the fact that it is practically impossible to obtain the stable structure of this protein for NMR studies in water, the compromise of using DMSO was justified because the ability to obtain high-quality NMR data for structure determination is well established using DMSO.

## 2. Materials and Methods

### 2.1. Protein Samples

AFGP fraction 8 (AFGP8) from the Arctic cod *Boreogadus saida* (AFGP8-BS) and the Antarctic notothenioid *Trematomus borchgrevinki* (AFGP8-TB) were prepared as previously described [5,6]. These fractions were also assayed previously for antifreeze activity by measuring thermal hysteresis using a capillary freezing–melting point technique [17]. The deuterated dimethyl sulfoxide (DMSO-d6) was purchased from Sigma Aldrich and was used as purchased. For both AFGP8 NMR samples, 18.0 mg of the AFGP8 was dissolved in 600 μL of DMSO-d6 in an Eppendorf tube, resulting in a concentration of 30.0 mg/mL. After the solutions were transferred into an NMR tube, the NMR tube was degassed (removing dissolved oxygen) and sealed to perform the NMR experiments. The disaccharide *beta-d-galactosyl-(1,3)-alpha-N-acetyl-d-galactosamine* (5 mg, 98% purity, CAS 3554-90-3, Cat No. A152000) was purchased from Toronto Research Chemicals (North York, ON, Canada) and was used as purchased. The NMR sample of the disaccharide was prepared in the same manner as that of the AFGP8 samples.

### 2.2. NMR Spectroscopy

The NMR experiments were performed using a Varian/Agilent VNMRS-400 MHz spectrometer (Palo Alto, California, USA) at the Chemistry Department of California State University, Fresno. All the experiments were performed using a One-NMR probe with a single axis (along *z*) pulsed field gradient. Additional experiments were performed at the University of California (UC) Davis NMR facility using a Varian 600-MHz triple resonance probe. The NMR experiments were performed at 298 K unless indicated otherwise. Two-dimensional (2D) experiments, including ^1^H-^1^H TOCSY (80 ms, 7.5 kHz, using Decoupling In the Presence of Scalar Interactions (DIPSI) [34]), ^1^H-^1^H NOESY, and ^1^H-^1^H Double Quantum Filtered COSY (DQFC), were acquired for sequential NMR assignments. Two-dimensional heteronuclear multiple quantum coherence (HMQC) was collected using the standard setup. Amide proton temperature coefficients were estimated from variable temperature ^1^H-^1^H TOCSY experiments over the temperature range 25–58 °C. Typically, the time domain data were collected with 2048 × 256 (*t*2 × *t*1) complex points in a phase-sensitive mode with a 2-s relaxation delay. Tetramethylsilane (TMS) was used as a chemical shift reference. All of the 2D NMR experiments were processed using NMRPipe [35] and analyzed using Sparky [36,37] and Mnova (www.mestrelab.com).

The diffusion coefficient of AFGP8 in DMSO was measured using the DOSY Bipolar Pulse Pair Stimulated Echo (DBPPSTE) sequence with convection compensation [38,39]. A diffusion delay of 0.2 s and a relaxation delay of 2 s were used. The gradient range used for each temperature was 500 to 26,500 DAC units in increments of 1000 DAC (1 DAC = 0.001744 G/cm), with 27 equal increments per experiment. The diffusion data were processed, and the corresponding hydrodynamic radius (*R_H_*) was estimated for each experiment using the program General NMR Analysis Toolbox (GNAT) [40,41,42]. From the estimated *R_H_* values, the radius of gyration was calculated using the scaling relation, *Rg* = 0.65 × *R_H_*, following Gaussian chain approximation.

### 2.3. Ensemble Characterization

The ensemble of representative structures was generated using a combination of NOESY cross-peaks (100 ms mixing time), chemical shifts, and the amide proton to alpha proton three-bond coupling constant (^3^*J_HNα_*). NOESY cross-peaks were calibrated within the framework of “combined assignment and dynamics algorithm for NMR applications” (CYANA) [43,44], and chemical shift values were converted into dihedral angle constraints using TALOS [45,46].

The glycosylated threonine was created and added to the software CYANA’s residue library as follows. A three-dimensional coordinate of the glycosylated threonine was created and optimized (Universal Force Field (UFF) with the steepest descent algorithm) using Avogadro (Version 1.1.1) [47]. Three-dimensional Cartesian coordinates were transferred into the CYANA residue library along with a total of 20 dihedral angles. The two pyranose rings did not have any dihedral angles defined, resulting in both pyranose rings remaining rigid in a (4–1) chair conformation. A copy of the library file is available upon request from the authors. The ^3^*J_HNα_* was determined from the 2D ^1^H-^1^H DQF-COSY spectrum. The ^3^*J_HNα_* was converted into dihedral angle constraints for the structure calculation. For AFGP8-BS, the following ^3^*J_HNα_* of alanine residues 5, 7, 8, 11, 13, and 14 and glycosylated threonine residues 3, 9, and 12 were also converted into dihedral angle constraints. For AFGP8-TB, the following ^3^*J_HNα_* of alanine residues 5 and 14 and glycosylated threonine residues 3, 6, 9, and 12 were used. The *J*-values were converted into dihedral angles with default settings within CYANA in the form of an allowed interval (φ1,φ2), with φ1 < φ2 < φ1 + 360°: The interval must not degenerate to a point.

Torsion angle dynamics implemented in CYANA generated an ensemble representation of NMR-based structures. Fifty-thousand random starting structures were annealed to an energy minimum within the given restraints. First, a set of 1000 lowest energy conformers were selected and referred to as the large ensemble of NMR structures. Of the 1000 conformations, the lowest energy was considered (128 in total) as a second set of the ensemble, referred to as a representative ensemble of NMR structures. Table S3 lists the total number of NOEs and upper limit distance constraints used for the structural calculation in CYANA. Table S3 also includes the RMSD of the 10 lowest energy structures. The mean structure of the 10 lowest energy structures was used for a Ramachandran plot using SwissPdb Viewer [48] (http://www.expasy.org/spdbv/). The large ensemble of NMR structures was used for a comparison to model structures generated using other methods (see below), and the representative ensemble of NMR structures was then used to define subclusters using NMRCLUST [49] implemented in Chimera [50].

A set of random structures for both AFGP8s was generated using CYANA. Using the random command in CYANA, 10,000 structures without any constraints were generated. Of the 10,000 structures, a random selection (without repeat) of 1000 structures was chosen and referred to as an ensemble of random structures. The three-dimensional structures are depicted using PyMOL (PyMOL Molecular Graphics System, Version 2.0 Schrödinger, LLC, New York, USA). The molecular structures were analyzed using in-house codes written using R [51] with established libraries such as Bio3D [52].

## 3. Results

### 3.1. Sequence-Specific Chemical Shift Assignments

**Figure 1:**
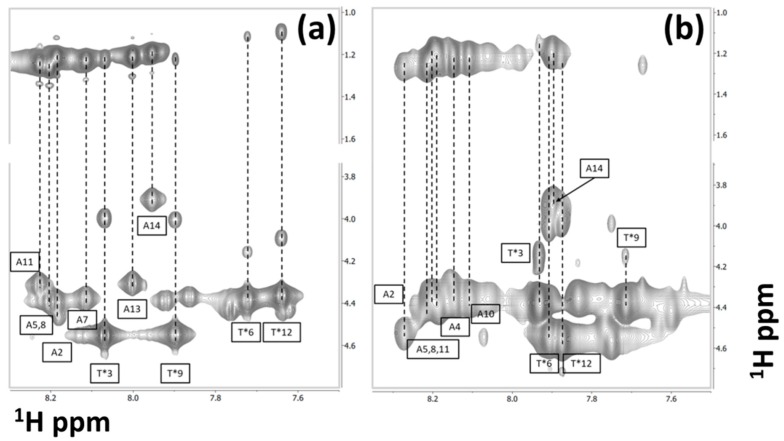
Sequence-specific chemical shift assignments of antifreeze glycoprotein fraction 8 (AFGP8). The 2D TOCSY NMR spectra of AFGP8-BS (AFGP8 from *Boreogadus saida*) (**a**) and AFGP8-TB (AFGP8 from *Trematomus borchgrevinki*) (**b**), showing the amide to alpha and methyl proton regions. The TOCSY patterns associated with all the amino acid residues of the primary sequence, except for the alanine residue at position 1 and proline residues at positions 4 and 10 in AFGP8-BS and positions 7 and 13 in AFGP8-TB.

The sequence-specific assignments of both AFGP8-BS and AFGP8-TB were performed using a combination of spectral data collected with the 400-MHz and 600-MHz NMR spectrometers. Distinct correlation patterns in the TOCSY spectra for the alanine and threonine residues were first utilized. As shown in Figure 1, for both AFGP8-BS (Figure 1a) and AFGP8-TB (Figure 1b), four threonine residues and seven alanine residues were identified. For both AFGP8 fractions, only seven out of the eight total alanine residues were identified, because the N-terminus alanine residue did not have an amide proton. The two proline residues at positions 4 and 10 for AFGP8-BS and positions 7 and 13 for AFGP8-TB had distinct patterns that showed up between the 2- and 5-ppm chemical shift region [53]. The resonance overlap between one of the beta protons and the two gamma protons of the proline residues was resolved using the DQFC spectrum. Upon completing the identification of individual backbone protons, a “NOESY–TOCSY” crosswalk was used to determine the sequence-specific assignments. Figures S1 and S2 show the completed sequence-specific assignments for AFGP8-BS and AFGP8-TB, respectively. The completed backbone chemical shifts for both AFGP8-BS and AFGP8-TB are listed in Table 1, and the measured three-bond coupling constants (^3^*J_HNα_*) are listed in Table S1.

### 3.2. Chemical Shift Assignments of the Carbohydrate Side Chains

**Figure 2:**
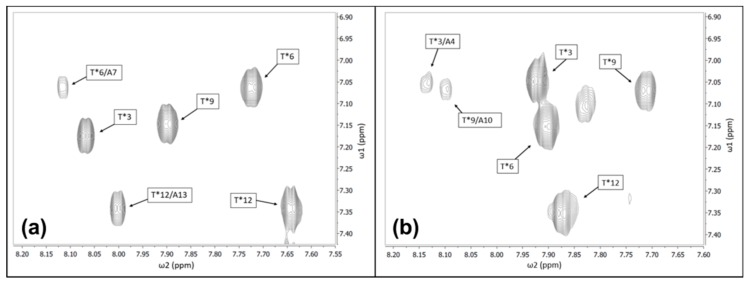
Characteristic nuclear Overhauser effect (NOE) between the backbone and side chain of Thr*. (**a**) AFGP8-BS and (**b**) AFGP8-TB 600-MHz 2D NOESY spectra focusing on the amide of the backbone to the amide of the disaccharide NOE correlation. The positions of the disaccharides and the threonine residues they were bonded to in the primary sequence are numbered in the black boxes.

To get a preliminary idea of the chemical shift distributions of the various protons, two-dimensional NMR experiments were performed on the disaccharide beta-d-galactosyl-(1,3)-alpha-*N*-acetyl-d-galactosamine. The amino acid residue positions of the four disaccharides, attached to the threonine residues, were determined by the NOE cross-peak between the amide proton of the disaccharides and the amide proton of the threonine residues, as shown in Figure 2. The chemical shifts of the first six-member ring (alpha-*N*-acetyl-d-galactosamine) of the disaccharide beta-d-galactosyl-(1,3)-alpha-*N*-acetyl-d-galactosamine were assigned starting from the amide proton of the *N*-acetyl group. The TOCSY pattern of the amide proton of each disaccharide showed four cross-peaks in the amide proton to alpha proton cross-peak regions. With the TOCSY spectrum alone, it was not clear which chemical shift belonged to the proton (C2 proton) adjacent to the amide proton. Using the DQFC spectrum, it was possible to determine the C2 proton chemical shift out of the four chemical shifts in the TOCSY spectrum. Starting from the amide proton, the rest of the protons on the first six-member ring of the disaccharide were assigned. The NOE correlation between the C3 proton of the first six-member ring and the C1′ proton of the second six-member ring was used to assist in assigning the proton chemical shifts of the second six-member ring. The rest of the chemical shift assignments of the ring protons, including the hydroxyl protons, were determined using a combination of TOCSY and DQFC data. Complete details about the assignment strategy can be found elsewhere [53]. The completed side-chain carbohydrate chemical shift assignments for both AFGP8-BS and AFGP8-TB are listed in Table 2, and the carbohydrate hydroxyl proton assignments are listed in Table S2.

### 3.3. Ensemble Characterization

Distance constraints were generated in CYANA using the integrated NOE volume obtained from the 2D NOESY spectrum of AFGP8-BS and AFGP8-TB. Summary numbers of distance constraints and their distributions are given in Table S3. A total of 131 distance constraints were assigned for AFGP8-BS. Of the 131 distance constraints, 64 resulted from an intra-residue NOE. For short-range and medium-range NOEs, 117 and 14 distance constraints were obtained. For AFGP8-TB, a total of 108 distance constraints were assigned. Of the 108 distance constraints, 53 resulted from an intra-residue NOE. For short-range and medium-range NOEs, 98 and 10 distance constraints were obtained. There was no long-range NOE detected between the amino acids, including the disaccharides, for both AFGP8-BS and AFGP8-TB. The italicized numbers in Table S3 next to the total NOE distance constraints represent the number of NOE correlations involving the disaccharide to the peptide backbone.

**Figure 3:**
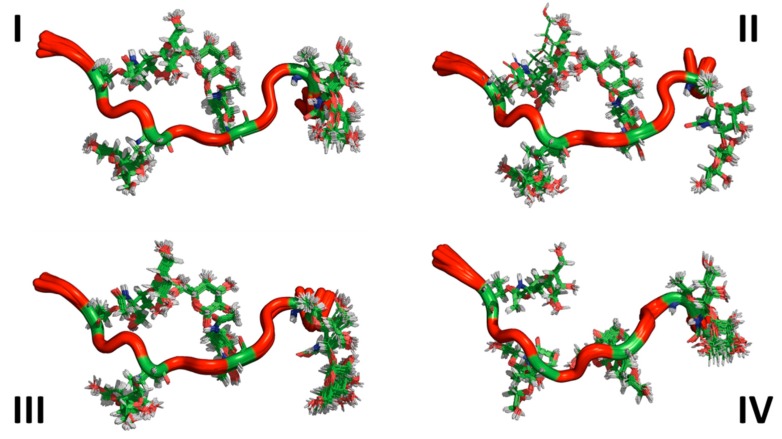
Clusters of substates in the representative ensemble NMR structures of AFGP8-BS. The top 4 clusters of the representative NMR structures of the total 128 structures. The structures are represented as cartoons, with the glycosylated threonine residue shown in lines. The number of structures in each cluster is Cluster I = 25, Cluster II = 22, Cluster III = 22, and Cluster IV = 11.

**Figure 4:**
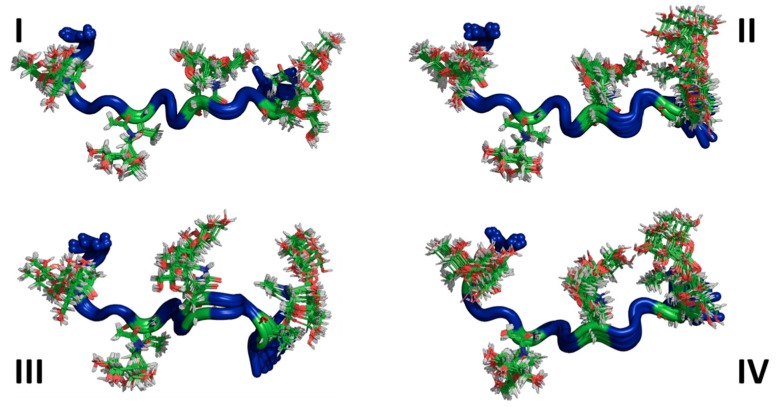
Clusters of sub-states in the representative ensemble NMR structures of AFGP8-TB. The top 4 clusters of the representative NMR structures of the total 128 structures. The structures are represented as cartoons, with the glycosylated threonine residues shown in lines. The number of structures in each cluster is Cluster I = 17, Cluster II = 17, Cluster III = 16, and Cluster IV = 14.

The sets of lowest energy structures identified by CYANA using the NMR-based constraints (also known as representative ensembles of NMR structures) for both AFGP8-BS and AFGP8-TB are shown in Figure 3 and Figure 4, respectively. Even within the representative 128 lowest energy structures, the NMRCLUST algorithm identified clusters with varying numbers of structures for both AFGP8s. For AFGP8-BS, a total of 16 clusters were identified, with the top 4 clusters (Figure 3) having 25, 22, 11, and 11 structures each. The other clusters had much fewer numbers of structures, ranging from nine to one. The clustering algorithm identified a similar number of clusters within AFGP8-TB (total of 15 clusters). The top 4 of the AFGP8-TB clusters contained 17, 17, 16, and 14 structures each, while the rest of the clusters varied from 8 structures to 1 structure each. For the AFGPs, the difference between the clusters was predominantly dominated by the relative orientation of the side-chain sugar moieties rather than the backbone structure.

**Figure 5:**
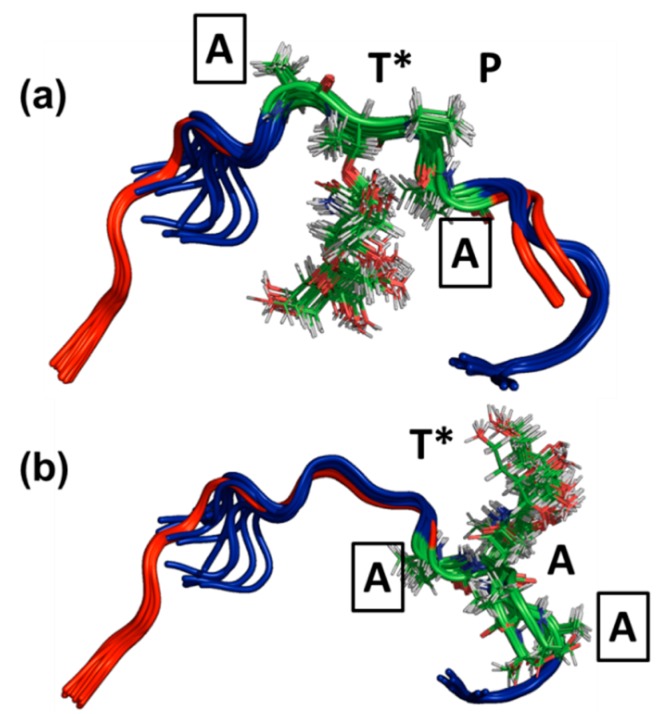
Similarity between the two AFGP8 proteins and motif definitions. (**a**) Local structural motif –Ala–Thr*–Pro–Ala– and (**b**) motif –Ala–Thr*–Ala–Ala–: The Ala residues defined as linkers (–Ala–) are boxed. The 10 lowest energy structures of BS (red) and TB (blue) are shown.

The primary sequence of AFGP8-BS between residues 1 and 11 and AFGP8-TB between residues 4 and 14 had the same sequential amino acid residue. Thus, Figure 5 shows the alignment of the BS (red) and TB (blue) structures, showing a significant overlap (RMSD = 1.74 Å) between the structures, particularly in the central portion. Figure 5 also identifies the local structural motifs, –Ala–Thr*–Pro–Ala– (Figure 5a) and –Ala–Thr*–Ala–Ala– (Figure 5b), with Ala preceding Thr* defined as a linker Ala that played a different structural role than a successive Ala. A preceding Ala was highly conserved in the definition of the motifs, while a successive Ala was replaced by Pro in a position-specific manner.

### 3.4. AFGP8-BS and AFGP8-TB Had a Distinct Ensemble in Solution State

**Figure 6:**
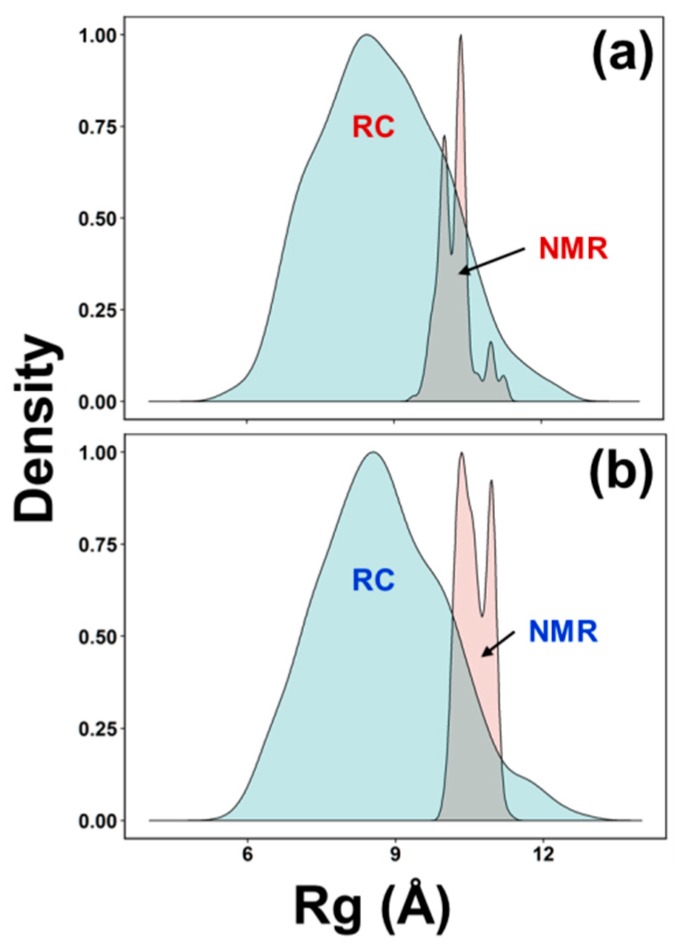
Comparison between the ensemble random coil and NMR-generated ensembles. (**a**) AFGP8-BS and (**b**) AFGP8-TB. The ensemble of the random coil (RC, light blue) and a large ensemble of NMR structures (NMR, light pink). The normalized density was calculated from an ensemble of 1000 structures each.

One of the objectives was to determine if AFGP8-BS and AFGP8-TB adopted a distinctly different ensemble of conformations from a typical random selection of conformations. A second, equally important question was, “Would the ensemble of conformations adopted by these peptides differ from each other?” The first question addresses if there is a preferential ensemble for AFGP8, while the second determines if the primary sequence influences the selection of the conformations. A comparison between the ensemble of conformations generated by assuming a random distribution Random Coil (RC), an ensemble of random conformations) and NMR-based modeling (NMR, a large ensemble of NMR structures) is presented in Figure 6. The density of distributions (normalized for each ensemble) of the radius of gyration (*Rg*) for AFGP8-BS (Figure 6a) and AFGP8-TB (Figure 6b) are plotted in Figure 6. Several notable features emerged: (a) The random coil ensembles of both AFGP8-BS and AFGP8-TB, as defined by the *Rg* values, were similar to each other; (b) the NMR-determined structural ensembles of AFGP8-BS and AFGP8-TB also had a similar range, with the distribution of *Rg* values being narrower than the random coil distribution; (c) and both AFGP8-BS and AFGP8-TB had two closely spaced subpopulations of structures, with the central population (~10.3 Å) being shared between them (Figure S4). These observations, in general, confirmed that AFGP8 did adopt a unique conformational ensemble, and the relative amino acid positioning did not have a notable difference in the overall distribution of the structures.

### 3.5. Size Estimations through Diffusion Coefficient Measures of AFGP8-BS and AFGP8-TB Suggested Comparable Compactness

**Figure 7:**
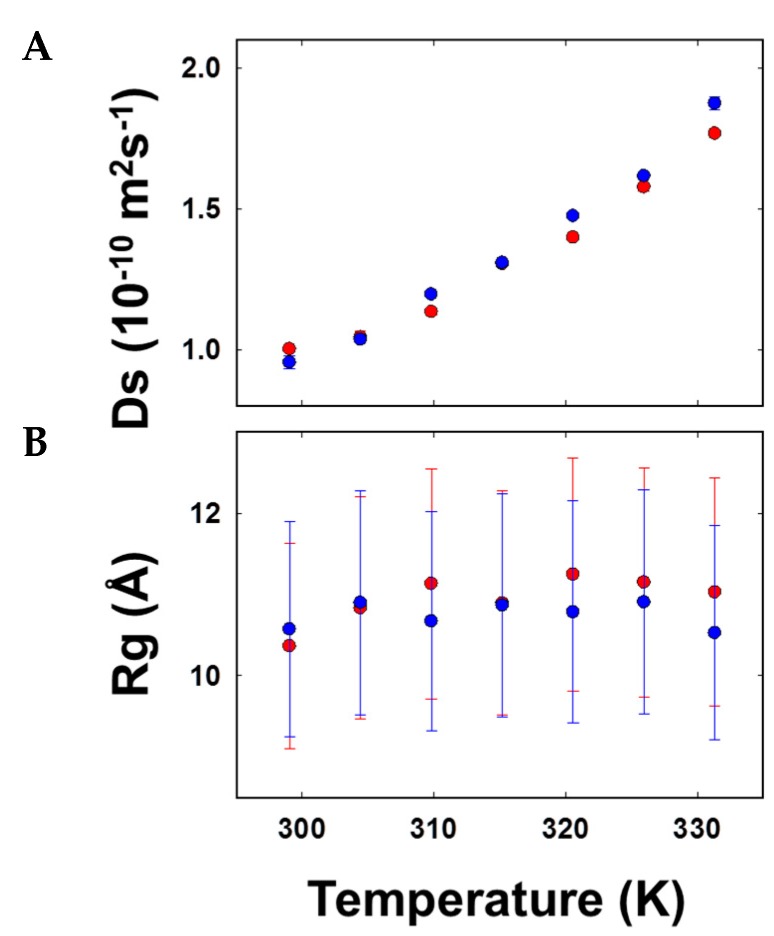
Molecular size estimation of AFGP8. (**A**) The plot of the translational diffusion coefficients of AFGP8-BS (red symbols) and AFGP8-TB (blue symbols) as a function of temperature. (**B**) *Rg* values of AFGP8-BS (red symbols) and AFGP8-TB (blue symbols) estimated by converting of the diffusion coefficient into the hydrodynamic radius.

The diffusion coefficient of AFGP8 was measured using NMR spectroscopy to determine if the relative proline residue positions altered the three-dimensional structure, in particular the compactness. Figure 7 shows the plot of the diffusion coefficient (Figure 7A) and the estimated *Rg* (Figure 7B) as a function of temperature for AFGP8-BS (red symbols) and AFGP8-TB (blue symbols). The diffusion coefficient of AFGP8-BS ranged from 1.02 ± 0.01 × 10^−10^ m^2^ s^−1^ to 1.77 ± 0.09 × 10^−10^ m^2^ s^−1^ over the temperature range of 25 to 58 °C. When adjusted for the viscosity of the solvent (DMSO) over the temperature range, the radius of the gyration value was estimated to be 10.95 ± 0.30 Å. The diffusion coefficient of AFGP8-TB in the same temperature range varied from 0.96 ± 0.03 × 10^−10^ m^2^ s^−1^ to 1.88 ± 0.04 × 10^−10^ m^2^ s^−1^, with the corresponding radius of gyration estimating to 10.75 ± 0.20 Å. The estimated *Rg* values of both AFGP8s using the diffusion coefficients closely resembled the *Rg* values estimated from the three-dimensional structures (Figure 6). In comparison to the diffusion coefficients of AFGP8-BS measured in water, the *Rg* values were smaller than the values in DMSO, confirming the role of hydration [54,55].

### 3.6. Role of Backbone to Side-Chain Hydrogen Bonding

**Figure 8:**
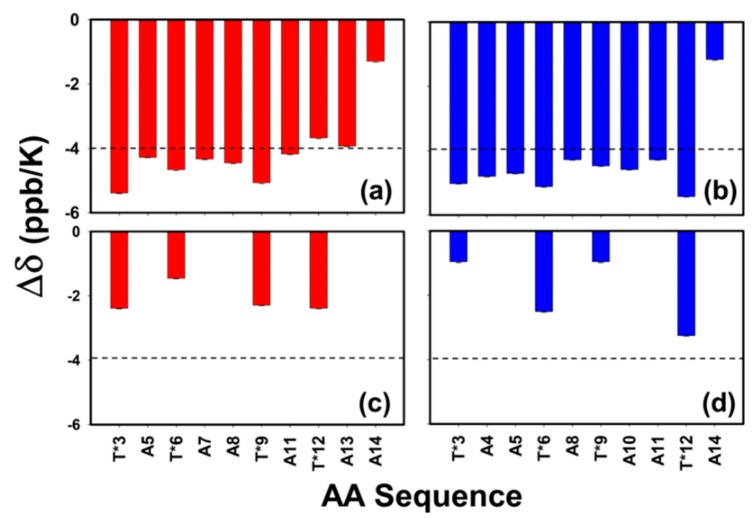
Amide proton temperature coefficients. Panels (**a**,**b**) correspond to the backbone amides of AFGP8-BS and AFGP8-TB, respectively. Panels (**c**,**d**) correspond to the side-chain amides of AFGP8-BS and AFGP8-TB, respectively. The horizontal dashed lines at the temperature coefficient of −4.00 ppm/°C suggests the cutoff for potential molecular hydrogen bonding formation.

With the three-dimensional structure determined, the hydrogen bonding potential of each amide proton was investigated. Amide proton temperature coefficients (rate of change of amide chemical shifts with temperature, Δδ) are good indicators of both intra- and intermolecular hydrogen bonding in peptides and proteins [56,57]. Figure 8 shows the rate of change in the chemical shift of the amide protons over the temperature range of 25–58 °C. The dashed line indicates the value of −4 ppb/°C, which was used to assess the potential of an amide proton being involved in hydrogen bonding [56,57]. The rate of change in the chemical shift (Δδ) of the backbone amide proton of AFGP8-BS (Figure 8a) and AFGP8-TB (Figure 8b) was near or below the prescribed cutoff value for the tendency of hydrogen bond formation (dashed lines of Figure 8a,b). In both AFGP8s, the C-terminus Ala residue showed a much lower amide temperature coefficient from the rest of the residues. Panels (c) and (d) in Figure 8 highlight the Δδ of the disaccharide amide proton of AFGP8-BS (c) and AFGP8-TB (d), showing a strong tendency to form hydrogen bonds, most likely to the backbone carbonyl of AFGP8. The trend in the amide coefficients of the side chain suggests that the disaccharide amide proton of the Thr* that was not adjacent to a proline residue had a higher value of Δδ than the ones that were adjacent to a proline residue, except for Thr*12 in AFGP8-BS. These results highlight the previous observation and suggestion by Mimura et al. on the role of carbohydrate side chain to backbone hydrogen bonding, which could be an essential feature of AFGPs [58].

## 4. Discussion

The solvent properties of water and DMSO have distinct differences. Though the structure of DMSO has some similarity to water, it is much more extended due to the S=O bond. DMSO is an aprotic solvent and is infinitely miscible in H_2_O. With the potential for hydrogen bonding, DMSO binds to water and becomes a space extender. However, due to its relative level of inability to H-bond, it has been seen that DMSO can act as a cryoprotectant [59,60]. For globular proteins that require stringent H-bonding with H_2_O for their structural identity, the use of DMSO as a substitute solvent denatures them. In contrast, in the case of intrinsically denatured proteins such as AFGPs, it may be that very little is different, except for the now-bestowed stability needed for these NMR experiments. Therefore, the local structures observed in our experiments may have been those reinforced by DMSO at the concentration used here.

A comprehensive characterization of the ensemble of structures presented by AFGP8-BS and AFGP8-TB in the solution state highlights several notable observations. With the ensemble of structures in the solution state for both AFGP8s being distinct and different from random coil structures (within the experimental conditions), the results strongly point out several key elements that may play an integral role in understanding the functional mechanism of AFGPs. These include confirming the role of the proline residues in the ensemble of conformation and defining the role of the structural motifs of AFGPs. These observations became possible because of our ability to determine the three-dimensional structures of both AFGP8s (originating from both *Boreogadus saida* (BS) and *Trematomus borchgrevinki* (TB)) in DMSO. The additional and equally valuable result includes the importance of hydrogen bonding between the carbohydrate side chain and the backbone of the protein. Regardless of this additional information leading to potential identification of local structural features, we consider the function of AFGPs to be dominated by their essence of being intrinsically disordered, a significant contrast when compared to their non-glycosylated counterparts, defined by the family of antifreeze proteins.

### 4.1. Integral Role of Proline Residues in the Short AFGPs

Differences in the NMR data were observed between AFGP8-BS and AFGP8-TB as a result of the sequential position of the proline residues. In general, the region containing a proline residue is more rigid compared to an alanine residue and introduces a kink in the structure. The proline residue may function as a steric hindrance barrier (restricting the precise movement of the disaccharides) that induces a local structural arrangement of the adjacent disaccharide, influencing the structural arrangement of the other disaccharides. In Figure 2, an inter-residue NOE correlation between the amide protons of the disaccharide and the amide proton of the alanine residue adjacent to the glycosylated threonine residue was observed. AFGP8-BS and AFGP8-TB both showed an inter-residue NOE correlation, but the NOE cross-intensities between Thr* and Ala were different depending on the presence of Pro preceding Thr*. The relative position of the proline residue might have been inducing a structural orientation in the adjacent glycosylated threonine residue. This induced local structural orientation promoted the next glycosylated threonine residue on the N-terminus side to take a similar orientation because any other orientation could have resulted in steric hindrance in the next local structural orientation, which would have been structurally unfavorable. Thus, the proline residues not only impacted, but also dictated, the orientation of the disaccharide units and hence the overall conformation of AFGP8.

In Figure 3 and Figure 4, the ensembles of the structures of AFGP8-BS and AFGP8-TB were different in comparison, as expected, with a difference in the position of the proline residues between the two AFGP8s. However, when aligning the 3D structures of the segment of amino acid residues 1–11 for AFGP8-BS and residues 4–14 for AFGP8-TB, where the amino acid residues occurred in the same sequential order in both AFGP8s, the disaccharides in each AFGP8 aligned right on top of each other, as shown in Figure 5. This indicated that the arrangement of the backbone might not have been as crucial as the arrangement of the disaccharides themselves. The proposal of AFGPs having a higher probability of adopting a polyproline II (PPII) secondary structure in solution was not conclusive, and it might have been due to the need for a certain arrangement of the disaccharides, not the backbone structure in terms of the Φ and Ψ values. By observing the overall 3D ensemble of structures of AFGP8-BS and AFGP8-TB, the disaccharides were arranged in an alternating pattern that did not expose the whole backbone on one side. This type of disaccharide orientation could be necessary for AFGPs to avoid proteolysis within the fish, as it is known that carbohydrates on proteins, such as mucins, prevent degradation by protease [61]. In addition, disordered regions and proteins are more prone to proteolysis, so this arrangement of the disaccharides, along with the dynamic nature of AFGPs, prevents protease from accessing the backbone for degradation of AFGPs, including the role of chaperone [62,63].

### 4.2. The Significance of Sequential Motifs

**Figure 9:**
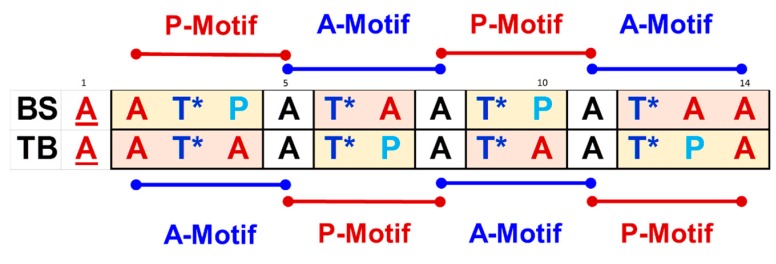
The primary sequence of antifreeze glycoprotein fraction 8 (AFGP8) from *Boreogadus saida* (BS) and *Trematomus borchgrevinki* (TB). The amino acid residues are color-coded as red (alanine, A), blue (glycosylated threonine, T*), and light blue (proline, P). Two consensus-repeating motifs are identified as –ATAA– (A-motif, blue horizontal lines) and –ATPA– (P-motif, red lines), with the overlapping alanine residue defined as the linker alanine residue shown in black. The second kind of alanine residue is shown in red. The linker alanine residue always precedes T*.

AFGP8-BS and AFGP8-TB are from two different natural sources. Each AFGP8 had two proline residues, but the proline residues were in a different position in the primary sequence (Figure 9). Regardless of the difference in the primary sequence, both AFGP8s performed the same antifreeze function, suggesting that there is subsequence juxtaposition that may play an important role. Both AFGP8s had the same number of amino acid residues and disaccharides. Between AFGP8-BS and AFGP8-TB, certain sequential motifs occurred the same number of times, and others occurred a different number of times. The tripeptide repeats (Ala–Ala–Thr*)_n_ are usually used to describe the primary sequence of AFGPs. From the structural similarities between AFGP8-BS and AFGP8-TB, we propose potentially two different motifs for AFGP8. Each of these motifs consists of two segments, as shown in Figure 9: (a) A P-motif defined by –Ala–Thr*–Pro–Ala– (shown as a red line, Figure 9) and (b) an A-motif defined by –Ala–Thr*–Ala–Ala– (shown as blue lines, Figure 9). In each of the motifs, the terminal Ala residue is overlapped over the segments (shown as black letters, Figure 9) and is defined as the linker Ala residue. The conformational and dynamic properties of AFGP8-TB (by Lane et al.) also proposed the same two segments [28]. In the current definition, we differentiate the two Ala residues, one that is part of the motif and a second that is the linker.

With these definitions, we suggest that combinations of A- and P-motifs would organize the local motifs in AFGPs. In defining the primary sequence of AFGP8-BS, Burcham et al. [64] determined that Pro–Ala (AA positions 7–8) is replaced by Ala at a ratio of 7:3 (~43%), while Ala–Ala (AA positions 10–11) is replaced by Pro at a ratio of 8:2 (~25%). Similarly, in defining the primary sequence of an arginine-containing AFGP from *Eleginus gracilis*, the authors determined that the ratio of Ala replacement at the Pro–Ala position is ~50% for the arginine-containing AFGP7 and that there is no Pro replacement at the Ala–Ala position for the arginine-containing AFGP8. These observations suggest that the frequency of alanine replacing proline is higher than proline replacing alanine in the primary sequence. Additionally, longer fractions of AFGPs do not contain proline residues [6]. Taken together, one could suggest that the higher fractions of AFGPs would contain more A-motif repeats than P-motifs, with less differentiation at the linker alanine.

### 4.3. N-Acetyl Functional Group of Disaccharides to Backbone Hydrogen Bonding

Using the temperature-dependent NMR data of the change in chemical shifts of the amide proton, Figure 8 shows the hydrogen bonding potential (<−4 ppb/K) calculated for each amide proton in AFGP8-BS and AFGP8-TB. The backbone amide proton of the glycosylated threonine adjacent to a proline seemed to be more sensitive to the temperature change. However, due to all the backbone amide protons potentially not being involved in hydrogen bonding (except for Ala14), it is difficult to conclude on the difference observed from the threonine residues. On the other hand, the *N*-acetyl amide proton of the disaccharides seemed to be potentially involved in hydrogen bonding, because the chemical shifts of these amide protons were less sensitive to temperature changes. The *N*-acetyl amide proton of the glycosylated threonine residues showed a similar trend in both AFGP8-BS and AFGP8-TB. There was lower hydrogen bonding potential for the *N*-acetyl amide proton of the glycosylated threonine residues adjacent to the proline residues, while the *N*-acetyl amide proton of the glycosylated threonine residues not adjacent to the proline residues had higher hydrogen bonding potential. Similar to the observation of the NOE correlation in Figure 2, Thr*12 of AFGP8-BS deviated from the expected trend. In AFGP8-BS, the *N*-acetyl amide proton of Thr*12 was more sensitive to temperature changes than the *N*-acetyl amide proton of Thr*6. The induced structural orientation of the disaccharides adjacent to the proline residues could have promoted the disaccharide on the N-terminus side to take a specific structural orientation. This seemed to be similar for the glycosylated threonine residues in both AFGP8-TB and AFGP8-BS, except for Thr*12 in AFGP8-BS. There was no proline residue or disaccharide on the C-terminus side of Thr*12: Thus the *N*-acetyl amide proton of Thr*12 had a stronger NOE correlation with the amino acid on its C-terminus. If there had been a proline residue or disaccharide in the C-terminus side of Thr*12, it would be expected that Thr*12 would not deviate from the trend observed for the glycosylated threonine residues.

From the 3D structure determined for AFGP8-BS and AFGP8-TB, no hydrogen bonding distance between 1.5 and 2.5 Å was measured between the *N*-acetyl amide protons and a backbone carbonyl. If AFGPs function as effectors, the disorder-to-order transition would occur upon binding to the ice surface. This disorder-to-order transition is expected to occur through the *N*-acetyl group of the disaccharide forming a hydrogen bond with the backbone, as proposed by Mimura et al. [58] in a study of mucin-type model glycopeptides. As a result, hydrogen-bonding distances would not be present until after the interaction between the ice surface and AFGPs. During the initial interaction between the unbounded AFGPs and the ice surface, the disaccharide on one of the motifs could take this orientation upon binding to the ice surface. Once one of the glycosylated threonine residues took this orientation, it would induce the adjacent glycosylated threonine residue to take the same orientation, and so forth: The same effect would occur for the other glycosylated threonine residues. It might not be required that all of the disaccharides of AFGPs bind onto the ice surface. There is a low probability that all of the disaccharides on the larger AFGP fractions would bind to the ice surface at the same time. Probably just a fraction of the disaccharides/motifs present in the AFGPs is required to bind onto the ice surface, acting as anchors to hold the AFGPs onto the ice surface while the unbound motifs remain disordered, thus allowing AFGPs to interact with the ice surface and the bulk water simultaneously.

Comparing the larger AFGP fractions to the smaller AFGP fractions at physiological concentrations, the larger AFGP fractions exhibited a higher degree of thermal hysteresis than the smaller AFGP fractions [64,65]. This was expected due to the larger AFGP fractions having a greater number of tripeptide repeats. At higher concentrations of AFGPs, an interesting phenomenon occurs: The smaller AFGP fractions exhibit a higher degree of thermal hysteresis compared to the larger AFGP fractions. The motif-based activity provides an alternate view on the activity of all the AFGPs. Within this model, a larger fraction of AFGPs is formed by linking “*n*” repeating motifs (a combination of A- and P-motifs). The P-motifs are essential in the smaller AFGPs to bind to the facets of the growing ice crystals, while the larger AFGPs inherently have larger coverage, with flexibility due to their fewer number of P-motifs. Therefore, at higher concentrations, the smaller fractions of AFGPs are more effective than the larger fractions of AFGPs. Consequently, the activity is related to the number of motifs rather than the concentration of the fractions.

The difference in thermal hysteresis properties between the smaller and larger AFGP fractions could have been because of the increase in the hydration shell/viscosity of water and the ability of the different AFGP fractions to bind onto the ice surface. At a physiological concentration of AFGPs, it is expected that most water molecules are hydration water molecules. Increasing the AFGP concentration only results in a slight increase in the number of hydration water molecules because only a small amount of water molecules left in the solution are bulk water molecules, resulting in a plateauing effect of the thermal hysteresis [3,64,65]. The ability of AFGPs to bind onto the ice surface is more crucial to an increase in the thermal hysteresis activity. A study by Burcham et al. [64,65] reported that the larger AFGP4 has an adsorption coefficient ~25 times that of the adsorption coefficient of AFGP8. However, at higher concentrations, AFGP8 exhibits a larger thermal hysteresis than the larger AFGP fractions do. It was speculated that the smaller AFGP fractions are less disordered than the larger AFGP fractions, thus allowing the smaller AFGP fractions to bind onto the ice surface more uniformly, consequently affecting adjacent ice binding sites less compared to the larger AFGP fractions.

Even though the higher molecular weight AFGP fractions exhibit a greater thermal hysteresis at physiological concentrations than the lower molecular weight AFGP fractions, the lower molecular weight AFGP components make up more than 75% of the AFGPs in fish blood serum [65]. This is likely due to the fish only having a finite amount of free water molecules within their system. Once all the water molecules are converted into hydration water molecules by AFGPs, adsorption onto the ice surface is the next step in increasing thermal hysteresis. Even though the kinetics study done by Burcham et al. [65] showed that the larger AFGP fractions have a better adsorption coefficient than the smaller AFGP fractions do, the smaller AFGP fractions are better at inhibiting ice crystal growth on the surface. This could explain why fish evolved to have the majority of their AFGP components be small AFGP fractions. In addition, the NMR data showed that the proline residues promoted some form of local structure on the motifs, whose structure influenced the adjacent motifs. While we assume that the proline residues made the smaller AFGP fractions intrinsically disordered by introducing kinks in the structure, unlike the synthetic AFGP analogs, the proline residues help promote local segment structure via the interaction of the *N*-acetyl amide proton with the backbone carbonyl. This interaction is crucial for the smaller AFGP fractions to bind onto the ice surface better than the larger AFGP fractions.

### 4.4. AFGPs as Intrinsically Disordered Proteins: Effectors and Entropic Chains

Tandem repeats are characteristic of intrinsically disordered regions or the peptides and proteins themselves being intrinsically disordered as a whole [66]. AFGPs consist of tandem repeats in their whole primary sequence. All fractions of AFGPs are considered to be disordered, not having a well-defined three-dimensional structure in a solution state [26]. The degree of the disorder depends on the number of tripeptide repeats, making the larger AFGPs with more tandem repeats more disordered than the smaller AFGPs with exclusively A-motifs [67]. The larger AFGPs have a more homogeneous repeat pattern of alanine and glycosylated threonine residues compared to the smaller fractions, the latter having some proline residues along with alanine and glycosylated threonine residues. It is expected that AFGPs would be highly disordered due to the near-perfect tandem repeat of the tripeptide Ala-Ala-Thr*. This disordered property is true for the larger AFGP fractions, such as AFGP1-5. Yet another possible structural influence of the proline residues is rendering the smaller AFGP fractions into intrinsically disordered peptides/proteins (IDPs). A study by Tachibana et al. [30] on a synthetic AFGP analog with three Ala-Ala-Thr* repeats showed that the three Ala-Ala-Thr* repeats formed a rigid solution structure mimicking a PPII secondary structure, with all of the disaccharides oriented toward the same side. The synthetic AFGP analogs function similarly to the natural AFGPs with respect to thermal hysteresis and restructuring the ice crystal shape. It is possible that the rigid solution structure of the synthetic AFGP analogs functions like natural AFGPs but cannot avoid proteolysis within the living organism. The rigid solution structure of synthetic AFGPs would expose the backbone to a protease that would degrade the synthetic AFGP analog in vivo. The proline residues prevent a rigid conformation of the smaller natural AFGP fractions by increasing the disorderliness of the overall conformation due to the kink in the structure that the proline residues induce in the smaller natural AFGP fractions. Such a structure does not allow the natural, smaller AFGP fractions to adopt a 3D conformation similar to the synthetic AFGP analogs. This might be the reason why proline residues are not common in the larger AFGP fractions, because the larger AFGP fractions are already highly disordered and the arrangement of the disaccharides does not expose the backbone to protease. The disordered state of AFGPs could be crucial for AFGPs to avoid protease degradation by mimicking a giant ball of carbohydrate.

Structural studies of AFGPs in a solution state have shown no significant change in the solution conformation of AFGPs even at temperatures close to water freezing. We hypothesize that AFGPs function as intrinsically disordered peptides/proteins (IDPs) [26]. AFGPs might function as effectors that make a disorder-to-order transition upon interacting with their binding partners, functioning as an entropic chain and a dynamic linker [66]. AFGPs would have a molecular functional feature in which AFGPs only undergo a disorder-to-order transition upon binding to the ice surface. As effectors do not have to make disorder-to-order transitions as a whole, effectors could have specific regions making a disorder-to-order transition. This would allow AFGPs to mimic an entropic chain as a dynamic linker that simultaneously interacts with both the ice surface and bulk water. It is possible for AFGPs to have these properties because AFGPs consist of tandem repeats of motifs (Figure 9).

### 4.5. Inferences about AFGP8 in Native Conditions: Water

In water, AFGPs have a larger hydration shell and increase the viscosity of water more compared to non-antifreeze proteins [32,68,69]. A study by Krishnan et al. [55] calculated the diffusion coefficient of AFGP fraction 8 (AFGP8) and several non-antifreeze proteins of different molecular weights using nuclear magnetic resonance (NMR) spectroscopy and hydrodynamic calculations. The hydrodynamic calculations were in good agreement with the NMR data, suggesting AFGP8 has a larger hydration volume compared to other non-antifreeze proteins. In recent studies using terahertz spectroscopy and molecular dynamics simulations, studies have shown that the hydration shell of AFGPs increases as a function of decreasing temperature [68,69]. The increase in the hydration shell of AFGPs suggests that at supercooled temperatures, AFGPs can perturb water molecules and their hydrogen-bonding network from a further distance than non-antifreeze proteins. The larger hydration shell of AFGPs compared to non-antifreeze proteins may be linked to the ability of AFGPs to increase the viscosity of water significantly [32]. An increase in the viscosity of water lowers the freezing point and decreases the probability of the bulk water molecules encountering ice crystals, thus inhibiting ice crystal growth [32,70]. The properties of increasing viscosity and larger hydration layers involve AFGPs in a solution state, but the restructuring of ice crystal morphology involves AFGPs interacting with the ice surface directly.

AFGPs’ ability to increase the viscosity of an aqueous solution as the temperature decreases prevents ice nucleation from occurring naturally, which could help explain the antifreeze mechanism. The long-range perturbation of water molecules and their hydrogen-bonding networks might be an essential characteristic of AFGPs’ antifreeze mechanism. Studies have reported AFGPs binding to the ice crystal surface [11,12,13] and have shown AFGPs being enveloped by the ice surface as the temperature drops below the hysteresis freezing point. In addition, the presence of superheated ice crystals above 0 °C indicates that AFGPs are interacting with the ice crystal surface, preventing the ice crystals from melting [8,9]. Interaction with the ice surface means that AFGPs must adopt a three-dimensional structure, if not as a whole then at least as short segmental motifs forming a local structure.

The secondary structure suggested for AFGPs in a solution state is that of a polyproline II helix (PPII). The basis for adopting the PPII secondary structure is the possibility of AFGPs aligning all of the disaccharides on one side, thus allowing AFGPs to form hydrophilic and hydrophobic sides. Computational studies have shown that AFGPs approach the ice surface via hydrophobic groups, such as the methyl groups presenting in alanine and threonine residues and the *N*-acetyl group of disaccharides [14,71]. The simulation of AFGPs approaching the ice surface via the hydrophilic groups and hydroxyl groups does not inhibit the ice surface from growing. During ice growth inhibition, AFGPs seem to have a higher probability of adopting the PPII secondary structure than the other possible secondary structures. One study also supported the reversible binding of AFGPs to the ice surface. On the contrary, a study by Meister et al. [72] showed that AFGPs bind irreversibly to the ice surface. It is still an open question whether the binding of AFGPs to the ice surface is reversible or irreversible, but it was suggested by Furukawa et al. [73] that the formation of the bipyramidal structures of ice crystals may require diverging adsorption mechanisms functioning on the different facets of ice.

A computational study supported the interaction of AFGPs with the ice surface via hydrophobic groups, but the hydrogen bonding nature of hexagonal ice (natural ice at an ambient condition) mimics that of a chair and boat conformation, which is the conformation that the disaccharides of AFGPs can take [74]. In addition, AFGPs have been shown to restructure the crystal shape of methyl α-d-mannopyranoside [75]. It seems that AFGPs tend to interact with crystals that have a structure similar to a chair and boat conformation, which the disaccharides can take. The interaction between AFGPs and the ice surface would be between the disaccharides of AFGPs and the ice surface. The disaccharides of AFGPs can mimic the structure of ice crystals, thus binding directly to the surface, while the other part of AFGPs that is not bound to the ice surface interacts with bulk water molecules. For the smaller AFGP fractions, there is a higher probability of most of the disaccharides being oriented on the same side, but for the larger AFGP fractions, such as AFGP1, it is highly improbable that all 55 disaccharides would face the same side. In their native state, AFGPs would act as effectors and fold upon binding. All of the disaccharides do not need to bind onto the ice surface, so AFGPs would act as a dynamic linker and interact with both the ice surface and water molecules, preventing direct contact for ice crystal growth.

## 5. Conclusions

Determination of the three-dimensional structure of antifreeze glycoproteins (AFGPs) in their native water solvent has proven to be very challenging, as has been evident from previous structural studies. Introducing AFGPs into a non-native solvent induced structural features that were determined using nuclear magnetic resonance (NMR) spectroscopy. A comparison of the three-dimensional structure determined by Lane et al. [28] in water to the current AFGP structure in DMSO confirmed that in the presence of water, AFGPs are highly dynamic overall compared to the more static structure determined in DMSO. This structural difference suggested that the native structure of AFGPs is not well defined and fits into the category of intrinsically disordered proteins. Using the short, conserved motifs present in both natural and synthetic AFGPs as the focus of structural studies is of significance due to the similarity between the structures of natural AFGP8 in DMSO and water and synthetic AFGP analogs in water. We suggest that perhaps the overall three-dimensional structure is not the most crucial feature, but the local structure that each conserved motif exhibits is of more importance. AFGPs might function as intrinsically disordered proteins, taking on local secondary structures and rendering themselves able to bind to multiple different ice-binding sites while simultaneously interacting with bulk water molecules.

If AFGPs function as intrinsically disordered proteins, AFGPs might be able to satisfy both proposed ice crystal inhibition models, ice crystal adsorption inhibition and long-range perturbation of water molecules. It has been shown with terahertz absorption spectroscopy and molecular dynamic simulation that the hydration layer of AFGPs increases with decreasing temperature [68,69]. In addition, AFGPs increase the viscosity of water upon cooling [32]. The combination of the mentioned properties could provide a good explanation of the often-asked questions, “Why do AFPs and AFGPs wait until ice crystal formation to start inhibiting ice crystal growth? Wouldn’t it be more effective to prevent ice crystals from forming at all?” Maybe, AFGPs inhibit ice crystal formation by increasing the viscosity of the solution and increasing its hydration layer to increase the range of protein-bulk water interaction. The increase in viscosity decreases the probability of the bulk water molecule encountering a heterogeneous growing ice crystal, thus inhibiting the ice crystal from reaching a critical size or radius preventing nucleation to occur spontaneously [32]. This inhibition of ice crystal formation with increasing viscosity is assisted by the longer-than-normal protein to bulk water interaction through the increase in the hydration layer. When the temperature gets low enough to promote spontaneous ice nucleation, AFGPs bind onto the ice crystal surface, thus matching the adsorption inhibition model. It would make sense that AFGPs need to be able to adopt multiple functional conformations, since the ice crystal surface would change upon restructuring, resulting in multiple different binding sites on the surface of the ice crystal. Within the thermal hysteresis gap, there exists both liquid water and small ice crystals. For AFGPs to function in both the solution and ice water interfaces, AFGPs must have multiple different functional conformations. The argument for AFGPs functioning as intrinsically disordered proteins satisfies multiple different functional conformations, since intrinsically disordered proteins can exist as multiple different conformations in solution while all conformations are still functional. The versatility of AFGPs could be the reason why two different species of fish from different polar regions evolved to produce nearly identical biological antifreeze, unlike the non-glycosylated antifreeze proteins that are structurally different in nature.

The compactness of the structural ensemble of AFGPs differed when their diffusion coefficient and three-dimensional structure were determined using NMR spectroscopy in water versus in DMSO. Using the short, conserved motifs present in both natural and synthetic AFGPs as the focus of structural studies might be of significance due to the similarity between the structures of natural AFGP8 in DMSO and in water and synthetic AFGP in water. Arguing thus, the overall three-dimensional structure might not be crucial, but the local structure that each conserved motif (Ala–Thr*–Pro–Ala or Ala–Thr*–Ala–Ala) takes could be more important. AFGPs could function as intrinsically disordered proteins, taking on local secondary structures and being able to bind to multiple different types of ice surfaces while simultaneously interacting with bulk water molecules.

## Figures and Tables

**Table 1:** Chemical shifts of AFGP8.

**Residue**	**(a) AFGP8 from *Boreogadus saida***								
	**H^N^**	**H^α^**	**H^β^**	**H^γ^**	**H^δ^**	**C^α^**	**C^β^**	**C^γ^**	**C^δ^**
A1	-	3.37	1.17	-	-	49.61	20.91	-	-
A2	8.18	4.47	1.22	-	-	47.51	18.28	-	-
T3*	8.07	4.55	4.00	1.23	-	54.63	74.19	18.29	-
P4	-	4.68	2.05; 1.88	1.90	3.63	58.63	29.10	23.94	46.74
A5	8.20	4.38	1.26	-	-	47.69	18.11	-	-
T6*	7.72	4.39	4.16	1.12	-	55.86	74.73	17.77	-
A7	8.12	4.38	1.23	-	-	47.68	18.28	-	-
A8	8.19	4.40	1.22	-	-	47.73	18.28	-	-
T9*	7.90	4.55	4.00	1.22	-	54.68	74.20	18.29	-
P10	-	4.70	2.05; 1.88	1.90	3.63	58.63	29.10	23.94	46.74
A11	8.23	4.31	1.24	-	-	47.82	18.20	-	-
T12*	7.64	4.36	4.09	1.09	-	55.34	74.12	17.80	-
A13	8.00	4.31	1.21	-	-	47.82	18.26	-	-
A14	7.95	3.91	1.20	-	-	48.65	18.30	-	-
**Residue**	**(b) AFGP8 *Pathogenia (Trematomus) borchgrevinki***								
	**H^N^**	**H^α^**	**H^β^**	**H^γ^**	**H^δ^**	**C^α^**	**C^β^**	**C^γ^**	**C^δ^**
A1	-	3.38	1.18	-	-	49.53	20.80	-	-
A2	8.21	4.53	1.25	-	-	47.55	18.01	-	-
T3*	7.92	4.38	4.16	1.13	-	55.87	74.82	18.29	-
A4	8.19	4.37	1.23	-	-	47.53	18.36	-	-
A5	8.18	4.41	1.21	-	-	47.79	17.68	-	-
T6*	7.90	4.55	4.01	1.22	-	54.58	74.02	18.64	-
P7	-	4.48	2.05; 1.88	1.88	3.62	58.62	28.98	24.22	46.82
A8	8.21	4.38	1.26	-	-	47.69	18.01	-	-
T9*	7.71	4.38	4.16	1.13	-	55.87	74.82	18.29	-
A10	8.10	4.47	1.22	-	-	47.53	18.69	-	-
A11	8.19	4.36	1.21	-	-	47.51	17.65	-	-
T12*	7.88	4.57	3.97	1.23	-	54.00	73.99	18.64	-
P13	-	4.38	2.01; 1.86	1.86	3.61	59.08	28.98	24.22	46.82
A14	7.86	3.87	1.20	-	-	48.65	18.30	-	-

**Table 2:** Chemical Shifts of the Disaccharides on AFGP8.

**Disaccharide**	**(a) AFGP8 from *Boreogadus saida***														
	**H^N^**	**H^NAc^**	**H^1^**	**H^2^**	**H^3^**	**H^4^**	**H^5^**	**H^6^**	**C^1^**	**C^2^**	**C^3^**	**C^4^**	**C^5^**	**C^6^**	**C^NAc^**
α3	7.18	1.83	4.88	4.18	3.64	3.90	3.71	3.47	98.5	47.8	77.8	67.7	71.2	60.4	22.8
α6	7.06	1.87	4.73	4.13	3.59	3.90	3.68	3.46	98.5	47.8	78.3	67.7	71.2	60.4	22.8
α9	7.15	1.83	4.88	4.13	3.61	3.90	3.71	3.47	98.5	47.8	78.2	67.7	71.2	60.4	22.8
α12	7.34	1.85	4.79	4.14	3.65	3.90	3.68	3.46	98.5	47.8	77.8	67.7	71.2	60.4	22.8
β3	-	-	4.24	3.31	3.27	3.62	3.34	3.53	104.1	70.7	72.8	67.9	75.1	60.4	-
β6	-	-	4.19	3.32	3.27	3.62	3.34	3.47	104.1	70.7	72.8	67.9	75.1	60.4	-
β9	-	-	4.21	3.32	3.27	3.62	3.34	3.53	104.1	70.7	72.8	67.9	75.1	60.4	-
β12	-	-	4.29	3.30	3.27	3.62	3.34	3.47	104.1	70.7	72.8	67.9	75.1	60.4	-
**Disaccharide**	**(b) AFGP8 from *Pathogenia (Trematomus) borchgrevinki***														
	**H^N^**	**H^NAc^**	**H^1^**	**H^2^**	**H^3^**	**H^4^**	**H^5^**	**H^6^**	**C^1^**	**C^2^**	**C^3^**	**C^4^**	**C^5^**	**C^6^**	**C^NAc^**
α3	7.05	1.87	4.72	4.15	3.62	3.90	3.68	3.47	98.5	47.9	78.2	67.7	71.3	60.4	22.8
α6	7.15	1.84	4.88	4.13	3.61	3.90	3.72	3.48	98.5	48.0	78.2	67.7	71.3	60.4	22.8
α9	7.07	1.87	4.72	4.14	3.58	3.90	3.68	3.47	98.5	47.9	78.3	67.7	71.3	60.4	22.8
α12	7.35	1.81	4.91	4.11	3.64	3.90	3.72	3.48	98.5	48.0	77.9	67.7	71.3	60.4	22.8
β3	-	-	4.23	3.28	3.27	3.62	3.34	3.47	104.0	70.6	72.8	67.9	75.1	60.4	-
β6	-	-	4.21	3.28	3.27	3.62	3.34	3.53	104.0	70.6	72.8	67.9	75.1	60.4	-
β9	-	-	4.20	3.28	3.27	3.62	3.34	3.57	104.0	70.6	72.8	67.9	75.1	60.4	-
β12	-	-	4.27	3.30	3.30	3.62	3.34	3.53	104.0	70.6	72.8	67.9	75.1	60.4	-

## Supplementary Materials

The following are available online at https://www.mdpi.com/2218-273X/9/6/235/s1, Figure S1: The TOCSY-NOESY crosswalk of AFGP8-BS, Figure S2: The TOCSY-NOESY crosswalk of AFGP8-TB, Figure S3: The distribution of distance constraints, Figure S4: Comparison of the NMR determined Rg values of AFGP8-BS and AFGP8-TB, Table S1: Three bond J-coupling constants (^3^J_HN__α_) of AFGP8, Table S2: Chemical shift assignments of the hydroxyl protons and Table S3: Number of NOEs and upper limit distance constraints.

## References

[1] Gordon M.S., Amdur B.H., Scholander P.F. (1962). Freezing Resistance in Some Northern Fishes. Biol. Bull..

[2] Scholander P.F., van Dam L., Kanwisher J.W., Hammel H.T., Gordon M.S. (1957). Supercooling and osmoregulation in arctic fish. J. Cell. Compar. Phys..

[3] DeVries A.L., Komatsu S.K., Feeney R.E. (1970). Chemical and physical properties of freezing point-depressing glycoproteins from Antarctic fishes. J. Biol. Chem..

[4] DeVries A.L., Wohlschlag D.E. (1969). Freezing resistance in some Antarctic fishes. Science.

[5] Osuga D.T., Ward F.C., Yeh Y., Feeney R.E. (1978). Cooperative Functioning between Antifreeze Glycoproteins. J. Biol. Chem..

[6] Feeney R.E., Yeh Y. (1978). Antifreeze proteins from fish bloods. Adv. Protein. Chem..

[7] Yeh Y., Feeney R.E. (1996). Antifreeze Proteins: Structures and Mechanisms of Function. Chem. Rev..

[8] Knight C.A., Devries A.L. (1989). Melting inhibition and superheating of ice by an antifreeze glycopeptide. Science.

[9] Cziko P.A., DeVries A.L., Evans C.W., Cheng C.H. (2014). Antifreeze protein-induced superheating of ice inside Antarctic notothenioid fishes inhibits melting during summer warming. Proc. Natl. Acad. Sci. USA.

[10] Bar Dolev M., Braslavsky I., Davies P.L. (2016). Ice-Binding Proteins and Their Function. Annu. Rev. Biochem..

[11] Raymond J.A., DeVries A.L. (1977). Adsorption inhibition as a mechanism of freezing resistance in polar fishes. Proc. Natl. Acad. Sci. USA.

[12] Davies P.L., Hew C.L. (1990). Biochemistry of fish antifreeze proteins. FASEB J..

[13] Hew C.L., Yang D.S. (1992). Protein interaction with ice. Eur. J. Biochem..

[14] Mochizuki K., Molinero V. (2018). Antifreeze Glycoproteins Bind Reversibly to Ice via Hydrophobic Groups. J. Am. Chem. Soc..

[15] Budke C., Dreyer A., Jaeger J., Gimpel K., Berkemeier T., Bonin A.S., Nagel L., Plattner C., DeVries A.L., Sewald N. (2014). Quantitative Efficacy Classification of Ice Recrystallization Inhibition Agents. Cryst. Growth Des..

[16] Davies P.L., Sykes B.D. (1997). Antifreeze proteins. Curr. Opin. Struct. Biol..

[17] De Vries A.L. (1986). Antifreeze glycopeptides and peptides: Interactions with ice and water. Methods Enzymol..

[18] Duman J.G. (2001). Antifreeze and ice nucleator proteins in terrestrial arthropods. Annu. Rev. Physiol..

[19] Feeney R.E., Burcham T.S., Yeh Y. (1986). Antifreeze glycoproteins from polar fish blood. Annu. Rev. Biophys. Biophys. Chem..

[20] Feeney R.E., Yeh Y. (1998). Antifreeze proteins: Current status and possible food uses. Trends Food Sci. Technol..

[21] Graether S.P., Sykes B.D. (2004). Cold survival in freeze-intolerant insects: The structure and function of beta-helical antifreeze proteins. Eur. J. Biochem..

[22] Harding M.M., Anderberg P.I., Haymet A.D.J. (2003). ‘Antifreeze’ glycoproteins from polar fish. Eur. J. Biochem..

[23] Harding M.M., Ward L.G., Haymet A.D.J. (1999). Type I ‘antifreeze’ proteins—Structure-activity studies and mechanisms of ice growth inhibition [Review]. Eur. J. Biochem..

[24] Fletcher G.L., Hew C.L., Davies P.L. (2001). Antifreeze proteins of teleost fishes. Annu. Rev. Physiol..

[25] Graether S.P. (2010). Biochemistry and Function of Antifreeze Proteins.

[26] Krishnan V.V., Yeh Y., Graether S.P. (2010). Structure and Functional Dynamics of Antifreeze Glycoproteins. Biochemistry and Function of Antifreeze Proteins.

[27] Urbanczyk M., Gora J., Latajka R., Sewald N. (2017). Antifreeze glycopeptides: From structure and activity studies to current approaches in chemical synthesis. Amino Acids.

[28] Lane A.N., Hays L.M., Feeney R.E., Crowe L.M., Crowe J.H. (1998). Conformational and dynamic properties of a 14 residue antifreeze glycopeptide from Antarctic cod. Protein Sci..

[29] Lane A.N., Hays L.M., Tsvetkova N., Feeney R.E., Crowe L.M., Crowe J.H. (2000). Comparison of the solution conformation and dynamics of antifreeze glycoproteins from Antarctic fish. Biophys. J..

[30] Tachibana Y., Fletcher G.L., Fujitani N., Tsuda S., Monde K., Nishimura S. (2004). Antifreeze glycoproteins: Elucidation of the structural motifs that are essential for antifreeze activity. Angew. Chem. (Int. Ed.).

[31] LeBel R.G., Goring D.A.I. (1962). Density, Viscosity, Refractive Index, and Hygroscopicity of Mixtures of Water and Dimethyl Sulfoxide. J. Chem. Eng. Data.

[32] Eto T.K., Rubinsky B. (1993). Antifreeze glycoproteins increase solution viscosity. Biochem. Biophys. Res. Commun..

[33] Heisel K.A., Krishnan V.V. (2014). NMR based solvent exchange experiments to understand the conformational preference of intrinsically disordered proteins using FG-nucleoporin peptide as a model. Biopolymers.

[34] Rucker S.P., Shaka A.J. (1989). Broadband homonuclear cross polarization in 2D NMR using DIPSI-2. Mol. Phys..

[35] Delaglio F., Grzesiek S., Vuister G.W., Zhu G., Pfeifer J., Bax A. (1995). NMRPipe: A multidimensional spectral processing system based on UNIX pipes. J. Biomol. NMR.

[36] Goddard T.D., Kneller D.G. (2008). SPARKY 3.

[37] Lee W., Tonelli M., Markley J.L. (2015). NMRFAM-SPARKY: Enhanced software for biomolecular NMR spectroscopy. Bioinformatics.

[38] Wu D., Chen A., Johnson C.S. (1995). An improved diffusion-ordered spectroscopy experiment incorporating bipolar-gradient pulses. J. Magn. Reson. Ser. A.

[39] Jerschow A., Müller N. (1997). Suppression of Convection Artifacts in Stimulated-Echo Diffusion Experiments. Double-Stimulated-Echo Experiments. J. Magn. Reson..

[40] Castanar L., Poggetto G.D., Colbourne A.A., Morris G.A., Nilsson M. (2018). The GNAT: A new tool for processing NMR data. Magn. Reson. Chem. MRC.

[41] Evans R., Dal Poggetto G., Nilsson M., Morris G.A. (2018). Improving the Interpretation of Small Molecule Diffusion Coefficients. Anal. Chem..

[42] Gierer A., Wirtz K. (1953). Molekulare Theorie der Mikroreibung. Z. Für Nat. A.

[43] Guntert P. (2004). Automated NMR structure calculation with CYANA. Methods Mol. Biol..

[44] Guntert P., Mumenthaler C., Wuthrich K. (1997). Torsion angle dynamics for NMR structure calculation with the new program DYANA. J. Mol. Biol..

[45] Shen Y., Bax A. (2015). Protein structural information derived from NMR chemical shift with the neural network program TALOS-N. Methods Mol. Biol..

[46] Shen Y., Delaglio F., Cornilescu G., Bax A. (2009). TALOS+: A hybrid method for predicting protein backbone torsion angles from NMR chemical shifts. J. Biomol. NMR.

[47] Hanwell M.D., Curtis D.E., Lonie D.C., Vandermeersch T., Zurek E., Hutchison G.R. (2012). Avogadro: An advanced semantic chemical editor, visualization, and analysis platform. J. Cheminform..

[48] Guex N., Peitsch M.C. (1997). SWISS-MODEL and the Swiss-Pdb Viewer: An environment for comparative protein modeling. Electrophoresis.

[49] Kelley L.A., Gardner S.P., Sutcliffe M.J. (1996). An automated approach for clustering an ensemble of NMR-derived protein structures into conformationally related subfamilies. Protein Eng..

[50] Pettersen E.F., Goddard T.D., Huang C.C., Couch G.S., Greenblatt D.M., Meng E.C., Ferrin T.E. (2004). UCSF Chimera—A visualization system for exploratory research and analysis. J. Comput. Chem..

[51] R Core Team (2013). R: A Language and Environment for Statistical Computing.

[52] Grant B.J., Rodrigues A.P., ElSawy K.M., McCammon J.A., Caves L.S. (2006). Bio3d: An R package for the comparative analysis of protein structures. Bioinformatics.

[53] Her C. (2018). Determination of the Solution Structure of Antifreeze Glycoprotein Fraction 8 (AFGP8) in Deuterated Dimethyl Sulfoxide (DMSO) Using Nuclear Magnetic Resonance (NMR) Spectroscopy.

[54] Inglis S.R., McGann M.J., Price W.S., Harding M.M. (2006). Diffusion NMR studies on fish antifreeze proteins and synthetic analogues. FEBS Lett..

[55] Krishnan V.V., Fink W.H., Feeney R.E., Yeh Y. (2004). Translational dynamics of antifreeze glycoprotein in supercooled water. Biophys. Chem..

[56] Iqbal M., Balaram P. (1982). Aggregation of apolar peptides in organic solvents. Concentration dependence of 1H-nmr parameters for peptide NH groups in 310 helical decapeptide fragment of suzukacillin. Biopolymers.

[57] Cierpicki T., Otlewski J. (2001). Amide proton temperature coefficients as hydrogen bond indicators in proteins. J. Biomol. NMR.

[58] Mimura Y., Yamamoto Y., Inoue Y., Chûjô R. (1992). NMR study of interaction between sugar and peptide moieties in mucin-type model glycopeptides. Int. J. Biol. Macromol..

[59] Mandumpal J.B., Kreck C.A., Mancera R.L. (2011). A molecular mechanism of solvent cryoprotection in aqueous DMSO solutions. Phys. Chem. Chem. Phys..

[60] Sydykov B., Oldenhof H., Sieme H., Wolkers W.F. (2018). Storage stability of liposomes stored at elevated subzero temperatures in DMSO/sucrose mixtures. PLoS ONE.

[61] Martinez-Saez N., Peregrina J.M., Corzana F. (2017). Principles of mucin structure: Implications for the rational design of cancer vaccines derived from MUC1-glycopeptides. Chem. Soc. Rev..

[62] Dunker A.K., Lawson J.D., Brown C.J., Williams R.M., Romero P., Oh J.S., Oldfield C.J., Campen A.M., Ratliff C.M., Hipps K.W. (2001). Intrinsically disordered protein. J. Mol. Graph. Model..

[63] Tompa P., Kovacs D. (2010). Intrinsically disordered chaperones in plants and animals. Biochem. Cell Biol. Biochim. Et Biol. Cell..

[64] Burcham T.S., Osuga D.T., Rao B.N., Bush C.A., Feeney R.E. (1986). Purification and primary sequences of the major arginine-containing antifreeze glycopeptides from the fish *Eleginus gracilis*. J. Biol. Chem..

[65] Burcham T.S., Osuga D.T., Yeh Y., Feeney R.E. (1986). A kinetic description of antifreeze glycoprotein activity. J. Biol. Chem..

[66] Van der Lee R., Buljan M., Lang B., Weatheritt R.J., Daughdrill G.W., Dunker A.K., Fuxreiter M., Gough J., Gsponer J., Jones D.T. (2014). Classification of intrinsically disordered regions and proteins. Chem. Rev..

[67] Jorda J., Kajava A.V., McPherson A. (2010). Protein Homorepeats: Sequences, Structures, Evolution, and Functions. Advances in Protein Chemistry and Structural Biology.

[68] Ebbinghaus S., Meister K., Born B., DeVries A.L., Gruebele M., Havenith M. (2010). Antifreeze glycoprotein activity correlates with long-range protein-water dynamics. J. Am. Chem. Soc..

[69] Mallajosyula S.S., Vanommeslaeghe K., MacKerell A.D. (2014). Perturbation of long-range water dynamics as the mechanism for the antifreeze activity of antifreeze glycoprotein. J. Phys. Chem. B.

[70] Wolfe J., Bryant G., Koster K.L. (2002). What is ‘unfreezable water’, how unfreezable is it, and how much is there?. Cryo Lett..

[71] Pandey P., Mallajosyula S.S. (2019). Elucidating the role of key structural motifs in antifreeze glycoproteins. Phys. Chem. Chem. Phys..

[72] Meister K., DeVries A.L., Bakker H.J., Drori R. (2018). Antifreeze Glycoproteins Bind Irreversibly to Ice. J. Am. Chem. Soc..

[73] Furukawa Y., Nagashima K., Nakatsubo S.I., Yoshizaki I., Tamaru H., Shimaoka T., Sone T., Yokoyama E., Zepeda S., Terasawa T. (2017). Oscillations and accelerations of ice crystal growth rates in microgravity in presence of antifreeze glycoprotein impurity in supercooled water. Sci. Rep..

[74] Malenkov G. (2009). Liquid water and ices: Understanding the structure and physical properties. J. Phys. Condens. Matter.

[75] Wang S., Wen X., DeVries A.L., Bagdagulyan Y., Morita A., Golen J.A., Duman J.G., Rheingold A.L. (2014). Molecular recognition of methyl alpha-D-mannopyranoside by antifreeze (glyco)proteins. J. Am. Chem. Soc..

