# Supplementary material for: The Ensemble of Conformations of Antifreeze Glycoproteins (AFGP8): A Study Using Nuclear Magnetic Resonance Spectroscopy
Source: Biomolecules. 2019 Jun 17;9(6):235. doi: 10.3390/biom9060235 (PMC6628104; doi:10.3390/biom9060235)
Supplement: Supplementary file 1 [file biomolecules-09-00235-s001.pdf]

Figure S1: The TOCSY-NOESY crosswalk of AFGP8-BS.

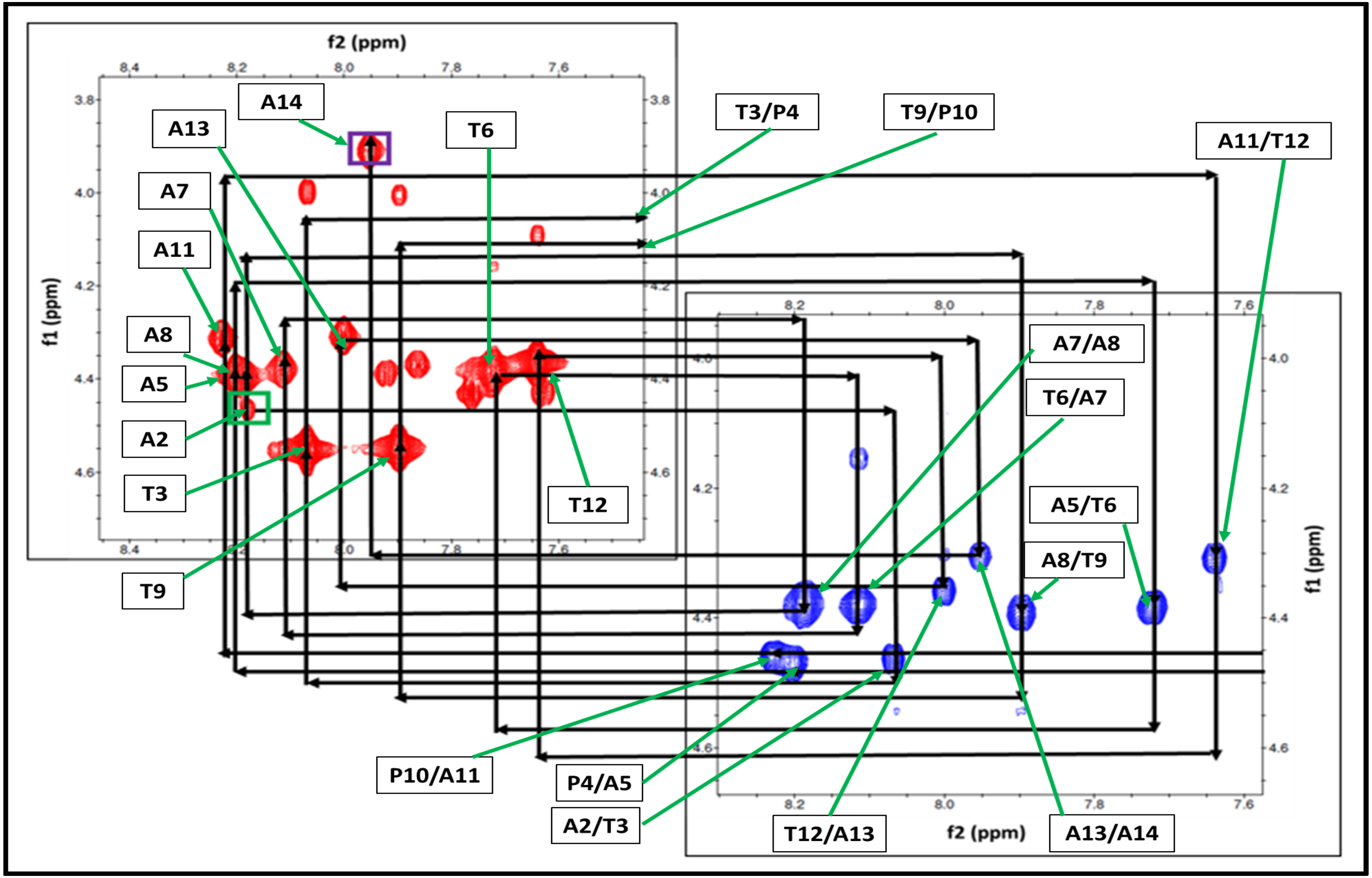

Figure S1

Figure S2: The TOCSY-NOESY crosswalk of AFGP8-TB.

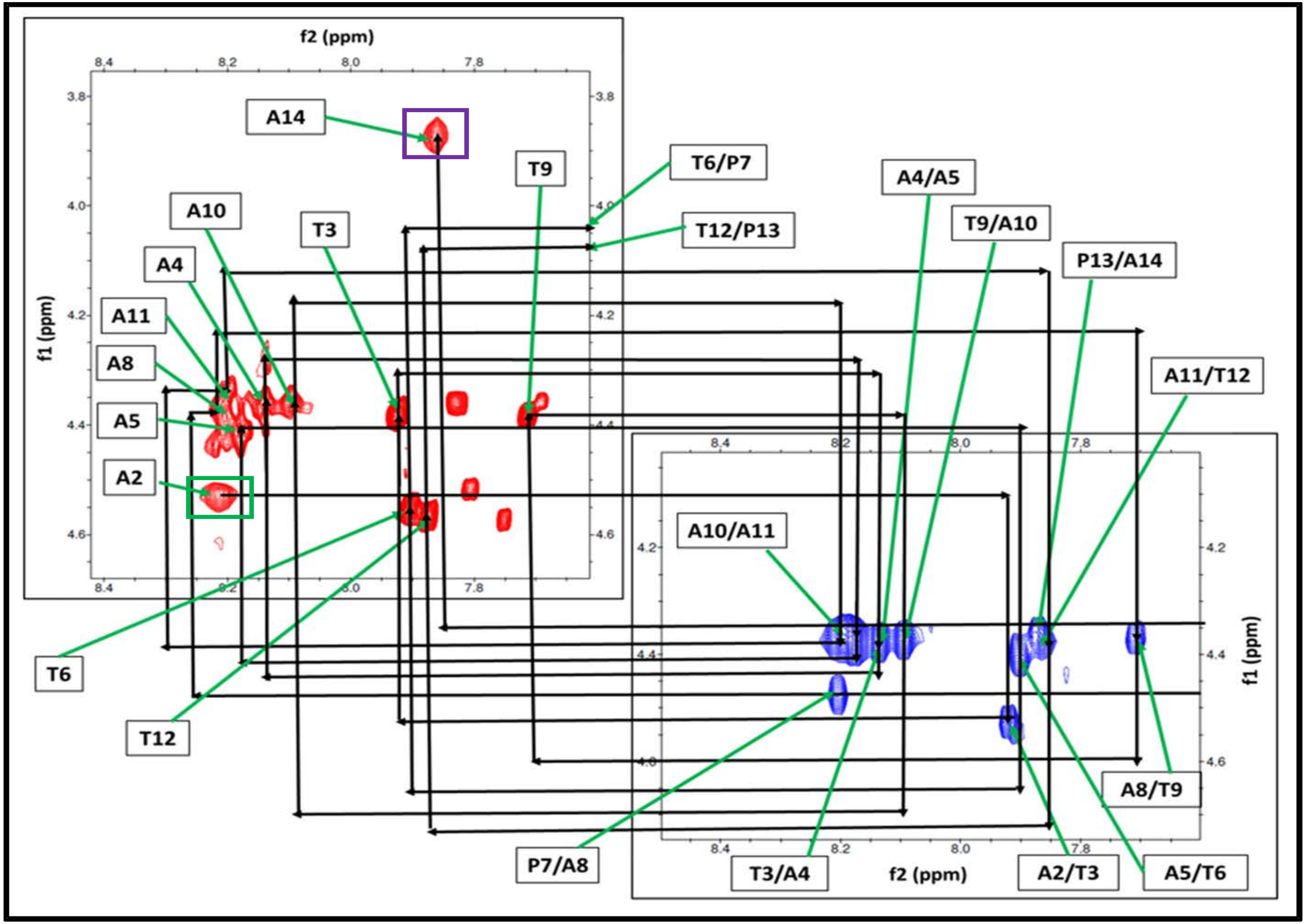

Figure S2

Figure S3: The distribution of distance constraints

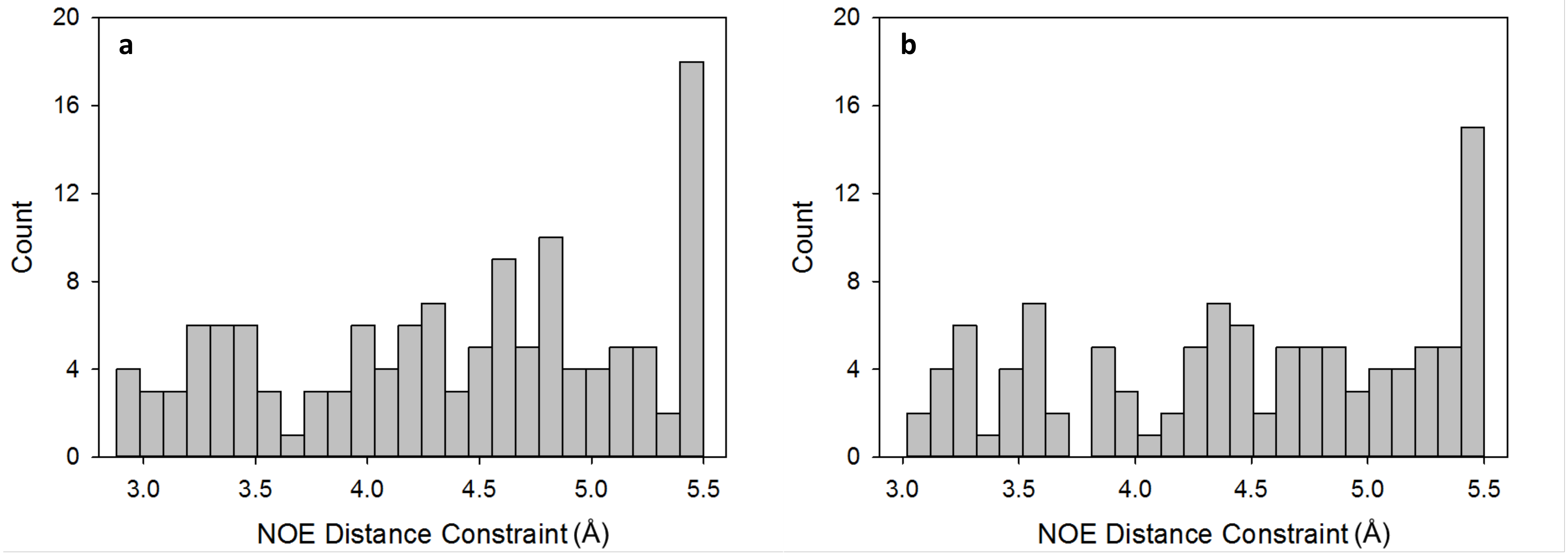

Figure S4: Comparison of the NMR determined Rg values of AFGP8-BS and AFGP8-TB

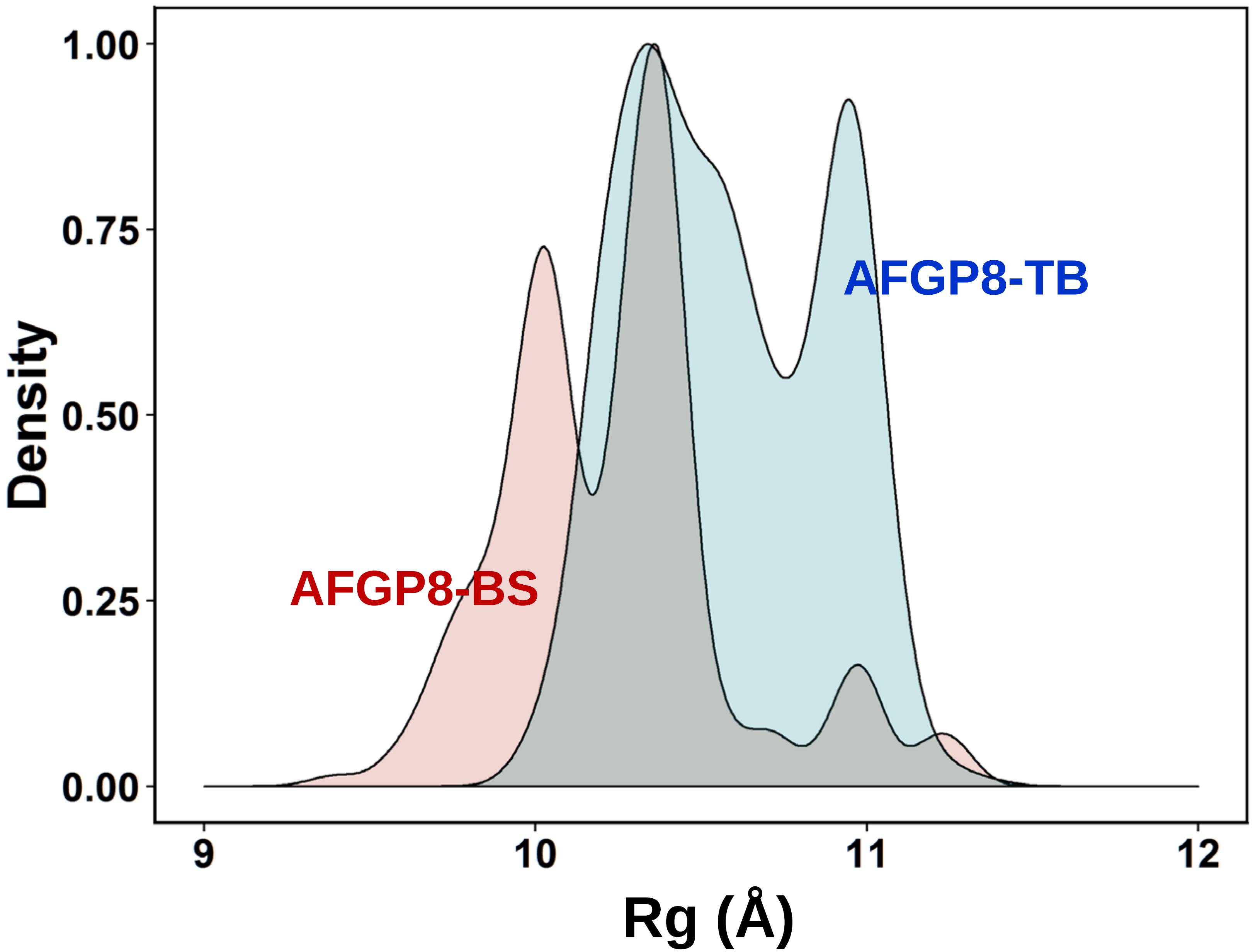

Figure S4

Table S1 – Three bond J-coupling constants ($^3J_{HN\alpha}$) of AFGP8

BS		TB	
Residue	$^3J_{HN\alpha}$ (Hz)	Residue	$^3J_{HN\alpha}$ (Hz)
T*3	9.0	T*3	9.6
A5	7.8	A5	8.4
A7	9.0	T*6	10.2
A8	8.4	T*9	10.8
T*9	10.2	T*12	9.6
A11	8.4	A14	8.4
T*12	9.6		
A13	8.4		
A14	8.4		

Table S2 – Chemical shift assignments of the hydroxyl protons

a) AFGP8 from *Boreogadus saida*				
Disaccharide	H^{O2}	H^{O3}	H^{O4}	H^{O6}
α3	-	-	4.45	4.63
α6	-	-	4.40	4.62
α9	-	-	4.42	4.63
α12	-	-	4.35	4.62
β3	-	4.80	4.39	3.53
β6	3.85	4.80	4.39	3.47
β9	3.77	4.80	4.37	3.53
β12	-	4.80	4.35	3.47

b) AFGP8 from *Pathogenia (Trematomus) borchgrevinki*				
Disaccharide	H^{O2}	H^{O3}	H^{O4}	H^{O6}
α3	-	-	4.43	4.62
α6	-	-	4.38	4.63
α9	-	-	4.45	4.62
α12	-	-	4.38	4.63
β3	-	4.79	4.33	4.59
β6	-	4.79	4.38	4.60
β9	-	4.79	4.38	4.59
β12	-	4.79	4.38	4.60

Table S3: Number of NOEs and upper limit distance constraints

	AFGP8-BS	AFGP8-TB
Total NOE Distant Constraint	131 (*61*)	108 (*49*)
Intraresidue	64	53
Sequential	53	45
Short-range	117	98
Medium-range	14	10
Long-range	0	0
Limit (to 2.99 Å)	4	0
Limit (3.00 to 3.99 Å)	39	31
Limit (4.00 to 4.99 Å)	54	44
Limit (5.00 to 5.99 Å)	34	33
Limit (6.00 to Å)	0	0
Average Backbone RMSD to Mean	0.50 ± 0.21	1.05 ± 0.19
Average Heavy Atom RMSD to Mean	0.58 ± 0.22	1.18 ± 0.23
